# Binocular disparity-based learning is retinotopically specific and independent of sleep

**DOI:** 10.1098/rstb.2019.0463

**Published:** 2020-04-06

**Authors:** Jens G. Klinzing, Lena Herbrik, Hendrikje Nienborg, Karsten Rauss

**Affiliations:** 1 Institute of Medical Psychology and Behavioral Neurobiology, University of Tübingen, 72076 Tübingen, Germany; 2 Centre for Integrative Neuroscience, University of Tübingen, 72076 Tübingen, Germany; 3 Princeton Neuroscience Institute, Princeton University, Princeton, NJ 08540, USA

**Keywords:** visual learning, memory, sleep, generalization, consolidation

## Abstract

Sleep supports the consolidation of recently encoded declarative and procedural memories. An important component of this effect is the repeated reactivation of neuronal ensemble activity elicited during memory encoding. For perceptual learning, however, sleep benefits have only been reported for specific tasks and it is not clear whether sleep targets low-level perceptual, higher-order temporal or attentional aspects of performance. Here, we employed a coarse binocular disparity discrimination task, known to rely on low-level stereoscopic vision. We show that human subjects improve over training and retain the same performance level across a 12-h retention period. Improvements do not generalize to other parts of the visual field and are unaffected by whether the retention period contains sleep or not. These results are compatible with the notion that behavioural improvements in binocular disparity discrimination do not additionally benefit from sleep when compared with the same time spent awake. We hypothesize that this might generalize to other strictly low-level perceptual tasks.

This article is part of the Theo Murphy meeting issue ‘Memory reactivation: replaying events past, present and future'.

## 1. Introduction

Sleep supports the consolidation of recently encoded memories, an effect that has been shown for numerous memory systems and underlying brain areas [1]. A critical mechanism of memory consolidation is the repeated reactivation of acquisition-associated neuronal activity patterns during post-learning sleep [2]. Sleep-associated consolidation results in the strengthening of new memory traces and their integration into existing memory networks. This entails a variety of qualitative changes, behaviourally expressed, for instance, as an improved ability to generalize learned information to new input.

In the visual domain, several studies showed benefits of sleep on improvements in the classical texture discrimination task (TDT) [3–9]. Improvements on the TDT are locally confined to trained regions of the visual field and the trained eye. This has been interpreted as evidence for the biological expression of visual learning on the lowest levels of the visual processing hierarchy, where input is still monocular and retinotopically organized [10]. Accordingly, a functional imaging study showed higher responses in the visual cortex to the TDT when stimuli were presented to the trained compared to the untrained eye [11]. Sleep after learning has been shown to aid the generalization of TDT improvements to the untrained eye [9]. Since this effect is hard to explain by local mechanisms alone, generalization might point to systems-level consolidation, possibly involving memory reactivation across widespread neuronal networks. Indeed, memory reactivation reflecting the same experience has been shown to be coordinated between hippocampus and visual cortex in rats [12]. Furthermore, recent research in mice has provided evidence for a role of the thalamus in the sleep-dependent consolidation of visually induced plasticity [13,14]. Animals were exposed to novel grating stimuli causing immediate neuronal response changes in the thalamic lateral geniculate nucleus. These changes were mirrored by the primary visual cortex only after the animals slept. During sleep, coherence between thalamic spikes and the cortical local field potential was increased and predicted the magnitude of later stimulus-specific responses in the visual cortex [14]. This suggests that the consolidation of plastic changes in the visual cortex might rely on the reactivation of visual information in the thalamocortical system during sleep.

To our knowledge, of the studies investigating sleep benefits on visual learning, all but one employed the classical TDT. Importantly, improvements on this task are assessed by a decrease of the time window between the target stimulus and a subsequent mask required for correct responses. One further study showed long-lasting benefits of sleep on the extraction of a prototype from abstract visual shapes [15]. For both these tasks, the higher performance was thus not necessarily derived from the perceptual component of the task. Here, we investigated the effect of sleep on a strictly perceptual task, namely coarse binocular disparity discrimination, which relies on the ability to use small differences in the image received by both eyes to assess depth. Since selectivity for binocular disparity in primates is first found in the primary visual cortex (for a review, see [16]), a task relying on binocular disparity allows us to isolate early visual cortical processing. Indeed, activity in visual area V2 is correlated with behaviour in this task [17,18], and has been shown to be causally involved in task performance in non-human primates (K Quinn and H Nienborg 2019, unpublished observations). Performance in binocular disparity-based tasks, including stereoacuity, fine and coarse disparity discrimination, has been shown to improve with training [19–22]. Potential effects of sleep could be realized as improvements compared to pre-retention levels [3,5,8], prevention of forgetting or deterioration over the retention period [5,23], or ability to generalize [9]. We investigated the effects of sleep on binocular disparity discrimination in an experimental paradigm that allowed us to detect and distinguish these different phenomena. Using sensitive within-subject contrasts, we compared the effects of wakefulness and sleep on post-retention performance, performance changes across the retention interval, and the ability to generalize to another location in the visual field. We hypothesized that subjects in the sleep condition would perform better than in the awake condition after the retention interval, either by showing a further increase in performance or by exhibiting less deterioration. We further predicted an increased ability to generalize these performance improvements across locations in the visual field following sleep.

## 2. Methods

### (a) Participants

For the main experiment, 17 subjects (10 female, 7 male; mean age ± s.d.: 23.06 ± 2.585, range: 19–28 years) were trained on a binocular disparity discrimination task (cf. [17]). The overall sample consisted of 23 subjects, six of which were excluded, either because they were unable to perceive disparity in the random-dot stereograms (*n* = 4), had insufficient sleep in the retention interval (*n* = 1), or were unable to maintain fixation (*n* = 1). All subjects showed a decimal visual acuity above 1.0, corresponding to a Snellen acuity of 20/20. Exclusion criteria were ongoing medication, health problems, medical interventions, night work, shift work, exam periods, stress-intense occupations during the three weeks prior to the experiment, or a history of psychiatric, neurological or sleep disorders. On experimental days, daytime naps, extensive physical exercise, as well as the intake of alcohol or caffeine were prohibited. The study was approved by the local ethics committee of the medical faculty of the University Tübingen. All subjects gave their written informed consent.

### (b) Visual task: coarse binocular disparity discrimination

**Figure 1.**
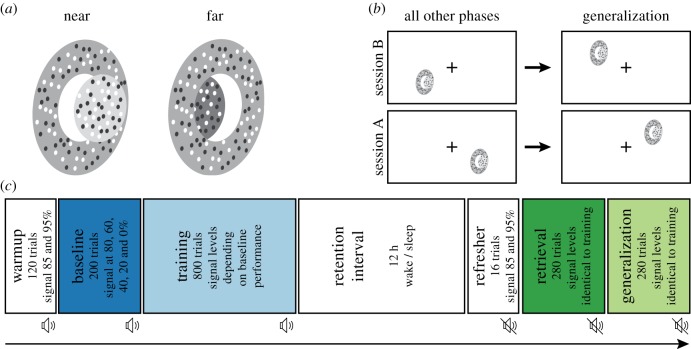
Visual learning task and experimental timeline. (*a*) Binocular disparity was used to differentiate two types of stimuli (near versus far). (*b*) The task was performed in the lower visual field, except for the Generalization test, which was performed in the matching upper visual field. Each subject participated in two sessions (A/B), the order of which was balanced. In one of the sessions, the task was performed mainly in the left visual field, in the other on the right. (*c*) In each session, three phases (Warmup, Baseline and Training) were followed by a retention interval and three further task phases (Refresher, Retrieval and Generalization). One of the two sessions started in the morning, the other one in the evening, such that the retention interval contained a period of night sleep or not. Auditory feedback (indicated by the speaker symbol) was given only before the retention period. Phases used to assess performance are colour-coded as in figure 2.

Participants performed a two-choice disparity discrimination task (figure 1*a*). They were asked to discriminate whether a central disk (3° diameter) was protruding (near) or receding (far) relative to a surrounding ring (1° wide). Both, ring and central disk, were circular dynamic random-dot stereograms, consisting of equal numbers of black and white dots (a new dot pattern was shown on each video-frame), comparable to those used previously [17,24]. Stimuli were shown on a back-projection screen using two projection design projectors (F21 DLP, 60 Hz, 1920 × 1080-pixel resolution, 70.5 cm image width, 225 cd m^−2^ mean luminance, linearized, 80 cm viewing distance) and passive linear polarizing filters with a relative tilt of 90°. Participants viewed the stimuli through passive linear polarizing filter glasses, such that each eye received light almost exclusively from one of the two projectors. This allowed us to show slightly different images to each eye, with disparity between the images resulting in the perception of depth. Only the central disk varied in disparity, whereas the ring was always shown at 0° disparity. On each video frame, all dots making up the central disk had the same disparity; the disparity value was changed randomly on each video frame (60 Hz) according to the probability mass distribution set for the stimulus. We manipulated task difficulty by changing the proportion of frames showing dots at the target disparity (defined as % signal strength). Target disparities (one value for ‘near' and one value for ‘far'), which determined the perceived distance between disk and ring, were easily detectable (i.e. well above the disparity detection threshold) in the absence of disparity noise and were held constant throughout the experiment (± 0.1°). For example, for a signal strength of 50% at the ‘near’ target disparity (−0.1°), 50% of the frames contained only the target disparity. On the remainder of the frames, disparities drawn from the noise distribution were shown. The disparity values of the noise were drawn from a uniform distribution (typically 11 values in 0.05° increments from −0.25° to 0.25°). For 0% signal trials, disparity values were drawn only from the noise distribution. Feedback on the 0% signal trials was randomized. Experimental control and stimuli presentation were achieved with custom written software (cf. [25]) using Matlab 2014a (MathWorks, Natick, USA) and Psychophysics Toolbox 3 [26,27].

Each trial was initiated by a button press. The stimulus was shown for 1.5 s with a horizontal offset (left or right depending on condition) of 3°. Vertical offset was set to 3° (top for Generalization test, bottom for all other phases). During stimulus presentation, a fixation cross was shown at the centre of the screen. The fixation cross disappeared together with the stimulus and two choice targets were shown above and below the previously fixated location for a maximum of 2 s. The choice target symbols were random-dot stereograms very similar to full signal stimuli, except that their diameter was smaller (2.2° disk diameter; 0.8° ring width). One of the targets showed a near stimulus, the other showed a far stimulus (100% signal strength). In a match-to-sample procedure, participants were instructed to pick the stimulus they had just seen using an up or down button press. Fixation on a centred cross was registered from stimulus onset until the start of the response time window, using an infrared eye tracker (Eyelink 1000, SR Research) at a sampling rate of 500 Hz.

### (c) Experimental timeline

All subjects participated in two sessions, which were scheduled three weeks apart. Each session consisted of six phases, three before and three after a retention period, respectively (figure 1*c*). Before the retention period, a Warmup phase introduced participants to the task by displaying stimuli with high signal strength (120 trials; signal at 85 or 95%). During a subsequent Baseline phase, we linearly sampled the entire range of signal strengths to assess the participant's performance (200 trials; signal at 80, 60, 40, 20 and 0%). In the Training phase, subjects were presented with lower (and therefore harder) signal strengths, depending on their performance during Baseline, with the addition of one easy, rarely sampled signal strength to assess the subject's lapse rate [28] (800 trials; signal strengths more densely sampled around 0, e.g. 90, 40, 20, 10 and 5%). After a Retention period, participants were briefly re-familiarized with the task (Refresher, 16 trials; signal at 85 or 95%). Afterwards, their performance was tested during Retrieval (280 trials; signal strengths identical to Training). During a subsequent Generalization test (280 trials; signal strengths identical to Training), transfer of retention performance was probed by presenting the stimulus in the upper visual field (figure 1*b*). During Warmup (after 40 initial exploration trials), Baseline and Refresher, trials were aborted in case of a fixation break. During other phases, fixation breaks were registered and trials were completed as usual, but data from these trials were discarded from analysis. The rate of correct fixations in these phases was generally very high (91.42 ± 6.18%). Feedback (correct versus incorrect response) was given during all phases preceding the retention interval. After the retention interval, feedback was disabled to minimize further visual learning.

All subjects started one of the two sessions in the morning, resulting in a daytime retention interval without sleep, and one of the sessions in the evening, resulting in a night-time retention interval including sleep for 7.41 ± 0.80 h (mean ± s.d.). Compliance to those instructions was verified and sleep duration measured using activity trackers with acceleration and light sensors (Actiwatch, Philips Respironics, Murrysville, USA). Sleep in the wake condition or sleep for less than 6 h in the sleep condition led to exclusion of the subject. Sleepiness at task onset was assessed using the Stanford Sleepiness Scale. Analysis showed a trend for subjects to exhibit an increase in sleepiness across the retention interval in the sleep condition and a decrease in sleepiness across the retention interval in the wake condition (interaction before/after × wake/sleep, *F*_1,16_ = 3.710, *p* = 0.072, *η*² = 0.084).

### (d) Data analysis

Only Baseline, Training, Retrieval and Generalization phases were analysed. We employed Bayesian inference for estimating psychometric functions by fitting a *β*-binomial model to the correct responses at each signal strength [29]. The following properties of the fitted cumulative Gaussian functions were used for analysing performance: pooled threshold (average of the signal strength required for 85% performance in the near/far direction), slope of the psychometric function (assessed at the signal strength leading to 50% correct responses), and bias (signal strength required for 50% correct responses). Only data for which our model explained greater than 80% of the variance were included (corresponding to 134 out of 136 datasets, for which the mean explained variance was 96.16%). Lapse rates were incorporated in the model and were generally very low (mean across subjects and experimental phases 1.84 ± 2.23%).

Grand average psychometric functions were generated by averaging model parameters across subjects. All extracted parameters were subjected to outlier rejection, following an interquartile range outlier rejection rule with a multiplier of 2.2 (lower threshold: Q1–2.2 × (Q3 − Q1); upper threshold: Q3 + 2.2 × (Q3–Q1)) [30]. Statistics were performed using JASP 0.10.0 (https://jasp-stats.org) and by calculating repeated measures ANOVAs incorporating the within-subject factors Phase (Baseline/Training/Retrieval/Generalization) and Wake/Sleep. To analyse effects of order instead of condition, a second ANOVA was conducted incorporating Phase and Session (First/Second). In another complementary analysis, we discarded the first 20 trials of the training phase and split the remaining trials into three blocks of 260 trials each. Also, the first 20 trials of subsequent phases were discarded, resulting in a homogeneous number of 260 analysed trials for Training 1–3, Retrieval and Generalization.

*Post hoc* tests were corrected using Holm's method. Only trials were analysed on which participants maintained fixation throughout the entire stimulus presentation. Results were Greenhouse-Geisser-corrected in case the assumption of sphericity was violated. Effect sizes are provided for significant tests (*η*² for ANOVA, Cohen's d for *post hoc* tests). Mann–Whitney *U* tests were used in case Levene's test showed a violation of the assumption of equality of variance. Additional Bayesian ANOVAs were conducted to quantify the evidence against effects for which classical tests returned non-significant results. Data were visualized using Python 3.7 and Seaborn 0.9.0.

## 3. Results

### (a) Task execution improves performance specifically at the trained location

**Figure 2.**
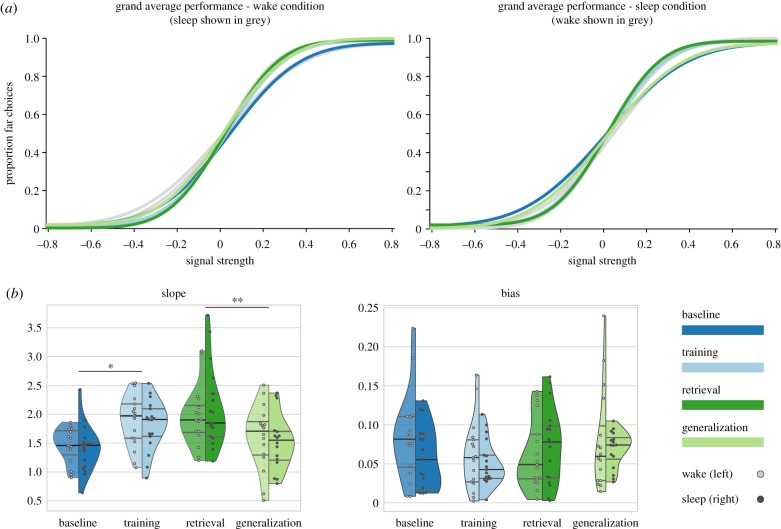
Performance of participants improved across task execution. (*a*) Grand average psychometric functions generated by fitting cumulative Gaussians to each subject's responses and averaging the resulting fitting parameters across subjects. For each condition (left: wake; right: sleep), Training and Retrieval exhibit a noticeably steeper slope compared to the other phases. (*b*) For each subject, condition and phase, model parameters were extracted to evaluate and compare performance. Performance improvements were reflected by significant main effects of Phase for the model parameter Slope (*p* < 0.001, other parameters reported in the main text), with performance improvements from Baseline to Training and performance deteriorations from Retrieval to Generalization. There was no significant change across phases in participants' bias and no significant difference between wake and sleep conditions. Violin plots show median, upper and lower quartile, extend to the most extreme values, and are vertically split to show the wake (left; light dots show individual subjects) and sleep (right; dark dots) conditions, respectively. **p < 0.01; *p < 0.05.

Similar to previous findings in a variant of the coarse disparity discrimination task [22], participants improved throughout the experiment, except for the Generalization phase (figure 2). During this last phase, stimuli appeared in the untrained upper visual field, resulting in a substantial drop in performance. This was indicated by main effects of Phase for the parameters Slope (*F*_3,39_ = 11.921, *p* < 0.001, *η*² = 0.203) and Threshold (*F*_3,42_ = 10.676, *p* < 0.001, *η*² = 0.171). *Post hoc* tests (Holm-corrected) for both Slope and Threshold revealed significant performance increases from Baseline to Training (Slope, *t* = 4.236, *p* = 0.004, *d* = 1.132 and Threshold, *t* = −4.227, *p* = 0.004, *d* = −1.091); insufficient evidence for a change over the retention period (*t* = 1.981, *p* = 0.138 and *t* = −1.843, *p* = 0.173, respectively); and a significant performance decrease from Retrieval to Generalization (*t* = −4.898, *p* = 0.001, *d* = −1.309 and *t* = 3.639, *p* = 0.011, *d* = 0.940). Furthermore, performance during Generalization did not significantly differ from Baseline (*t* = 0.856, *p* = 0.407 and *t* = −0.689, *p* = 0.502). For a detailed visualization of changes in performance during Training, see electronic supplementary material, figure S1. For the parameter Bias, quantifying the observer's tendency towards one or the other response, our results are most compatible with no important effect of Phase (*F*_2.020,26.255_ = 1.195, *p* = 0.319). When analysing the percentage of correct responses, only data from the Training, Retrieval and Generalization phases were included, since task difficulty was personalized after the Baseline phase. Similar to Slope and Threshold, we observed a main effect of Phase (*F*_2,32_ = 25.860, *p* < 0.001, *η*² = 0.154), and *post hoc* tests indicated no significant change across the retention period (*t* = 1.357, *p* = 0.194), but a drop in performance during Generalization (*t* = −5.304, *p* < 0.001, *d* = −1.286).

In the analysis based on session order, subjects descriptively performed better in their second session, as tentatively suggested by trends for main effects of Session for Slope (*F*_1,14_ = 2.665, *p* = 0.125, *η*² = 0.024) and Threshold (*F*_1,14_ = 3.899, *p* = 0.068, *η*² = 0.036), in the absence of a significant Session × Phase interaction (*F*_3,42_ = 0.137, *p* = 0.937 and *F*_3,42_ = 1.070, *p* = 0.372).

### (b) Task performance and its generalization are not affected by sleep

None of the psychophysical parameters was affected by sleep, as indicated by an absence of significant main or interaction effects (Slope, main effect Wake/Sleep *F*_1,13_ = 0.100, *p* = 0.757 and interaction Wake/Sleep × Phase, *F*_3,39_ = 1.194, *p* = 0.325; Threshold, *F*_1,14_ = 0.523, *p* = 0.481 and *F*_3,42_ = 2.106, *p* = 0.114; Bias, *F*_1,13_ = 0.211, *p* = 0.653 and *F*_3,39_ = 0.716, *p* = 0.548; Per cent correct, *F*_1,16_ = 0.459, *p* = 0.508 and *F*_2,32_ = 0.322, *p* = 0.727). To substantiate these null results, we conducted equivalent Bayesian repeated measures ANOVAs using default priors in JASP (*r* scale fixed effects = 0.5, *r* scale random effects = 1). For parameters significantly affected by Phase (i.e. Slope, Threshold and Per cent correct), we included this factor into the null model. Results indicated strong evidence against the full model with main and interaction effects of Sleep/Wake for all parameters (Slope, BF_01_ > 22; Threshold, BF_01_ > 10; Bias, BF_01_ > 98; Per cent correct, BF_01_ > 22). This was largely supported by analyses of individual effects, with strong evidence against the inclusion of the interaction term for Slope (BF_excl_ > 13), Bias (BF_excl_ > 40), and Per cent correct (BF_excl_ > 13); and moderate evidence against the interaction term for Threshold (BF_excl_ > 6.9). For a more detailed investigation of performance changes across the retention interval, we directly compared Slope and Threshold during the third Training block (last 260 trials) and Retrieval (electronic supplementary material, figure S1). This comparison did not yield a significant interaction of Wake/Sleep × Phase (Slope, *F*_1,15_ = 2.313, *p* = 0.149; Threshold *F*_1,16_ = 0.002, *p* = 0.969), again arguing against a specific sleep benefit.

To rule out that the interval of three weeks between sessions was insufficient to prevent carry-over effects, we added a categorical covariate ‘sleep first' to the repeated measurements ANOVA. Inclusion of the covariate did not substantially change the results (Slope, main effect Wake/Sleep *F*_1,12_ = 1.128, *p* = 0.309, interaction Wake/Sleep × Phase, *F*_3,36_ = 0.225, *p* = 0.879, and between-subject effect of sleep first, *F*_1,12_ = 0.188, *p* = 0.672; Threshold, *F*_1,13_ = 0.706, *p* = 0.416, *F*_3,39_ = 0.648, *p* = 0.589 and *F*_1,13_ = 0.828, *p* = 0.379). We additionally analysed generalization separately for ‘Sleep first' (*n* = 9) and ‘Wake first’ (*n* = 8) subjects. Performance changes from Retrieval to Generalization in their respective first sessions did not significantly differ between these groups (Slope, *t* = 1.428, *p* = 0.175; Threshold, *t* = −0.265, *p* = 0.795), again arguing against the idea that sleep improves generalization of discrimination performance in this task to different visual field locations.

Visual processing in primates is known to differ in several aspects between the upper and lower visual field [31,32]. These differences could potentially mask generalization of the learned skill across the horizontal meridian. We therefore analysed performance changes from the first to the second session in more depth. Comparison of the respective Baseline conditions revealed that while performance was slightly but not significantly improved for session 2 (*p* = 0.298, Wilcoxon test), Baseline performance in session 2 did not reach the level of the Retrieval phase in session 1, although the difference did not reach significance (*p* = 0.084, Wilcoxon). Moreover, we analysed whether performance changes from Retrieval in the first session to Baseline in the second session differed between the ‘Sleep first' and ‘Wake first' groups, which would point to an improved generalization across the vertical meridian. Again, results were most compatible with no important difference between the ‘Sleep first' and ‘Wake first' groups (Slope, Mann–Whitney *U* = 32.000, *p* = 1.000; Threshold, *t* = 0.453, *p* = 0.657).

## 4. Discussion

Subjects successfully improved their ability to differentiate stimuli based on binocular disparity signals. These improvements did not generalize across the horizontal or vertical visual meridian. Both initial learning and its generalization were unaffected by a full night of sleep when compared to a full day of wakefulness. These results were obtained in the context of a powerful within-subjects design, were confirmed by additional analyses within and across sessions, and are therefore robust. This was corroborated by Bayesian analyses providing strong quantitative evidence against effects of sleep on performance. Our analysis of cross-session vertical generalization assumed that effects of one night of sleep after training would still be detectable after three weeks. While this may seem unlikely, there is evidence for extremely long-lasting effects of sleep right after training on a visual skill [15]. Taken together, we believe the present data convincingly demonstrate the absence of an important beneficial effect of sleep on improvements in binocular disparity discrimination.

Previous studies have shown sleep-associated consolidation for visual skills (i.e. orientation discrimination [3–9] and gist abstraction [15]). These skills differ in important aspects from the learning in our coarse disparity discrimination task, which required extracting the target disparities embedded in time-varying disparity noise and might thus rely on different neuronal mechanisms. In particular, in the texture discrimination paradigm, task difficulty is not manipulated by altering the stimulus itself. The discriminated texture and its presentation time window remain identical while difficulty is manipulated by shortening the time between texture offset and subsequent onset of a mask. Therefore, only temporal aspects of the task contribute to changes in difficulty. Training subjects on these temporal details beforehand has been shown to minimize further improvements on the TDT [33] and improvements on the task have consequently been attributed mainly to temporal learning. This type of learning may thus result to some extent from optimizations in the temporal allocation of attention [34], in addition to genuine low-level visual learning. These differences in the neuronal mechanisms of learning may be the source of conflicting results concerning their sleep-associated consolidation. Fine-tuned attentional allocation, presumably resulting from complex interactions of thalamic and neocortical structures [35], has been proposed to be an integral driver of sleep-associated memory consolidation [2]. Attention may inform subsequent consolidation by guiding cross-regional reactivation of neuronal firing patterns at the most appropriate level of processing [36]. Such systems-level consolidation may result in the generalization of learning to other parts of the visual field. If low-level visual learning, as investigated in the present study, is largely independent of temporal attentional allocation, this may result in no or only local reactivations that do not support generalization.

An alternative explanation for the lack of a benefit of sleep on disparity learning might come from its inherently binocular nature. One earlier study on the effects of sleep on the generalization of visual skills reported an interocular transfer of monocular improvements in texture discrimination [9]. Such generalization might be achieved when visual stimuli are reactivated in lower-level areas and communicated to higher-level neuronal populations. In cases for which performance improvements are achieved most effectively at earlier stages of visual processing, the reactivated information might then be fed back to lower-level neurons, with diverse inputs and receptive fields, resulting in plasticity compatible with the reported generalization. By contrast, in the present paradigm, binocular information is the only visual parameter for optimizing performance. Improvements might, therefore, be coded at an intermediate level of the visual hierarchy, too high to trigger low-level reactivations, and too low and non-declarative to be triggered by reactivations in the hippocampus or frontal cortex.

Potential confounds in our data may arise from the training regime. For example, one might suspect that the total of 1120 trials performed before the retention period trained subjects to near-optimal performance, which might have obscured further sleep-dependent performance enhancements. However, unpublished observations (JG Klinzing, H Nienborg, K Rauss 2019) from a single subject trained over four sessions and a total of 3200 trials suggest that performance in this task can improve much beyond the level of the participants in this study. On a related note, it has been shown that the introduction of salient dummy trials may affect generalization in perceptual tasks [37]. In the present study, easy trials were added in order to account for lapse rates when fitting the psychometric function. Estimating lapse rates requires performance data at a signal strength at which non-optimal performance is not due to a perceptual cause [28]. Beyond this, easier trials are likely to raise motivation by allowing a sense of achievement despite progressively increasing task difficulty. Given the low number of these trials and their mean signal strength of 72% (which is substantially less than the warmup trials at 85 and 95% signal strength), it seems unlikely that they exerted a substantial effect on learning and performance. If anything, based on the above study [37], interleaved easy trials should favour generalization instead of preventing it.

The present paradigm differs from previous studies in another important aspect in that it relies on dynamically changing stimuli. To our knowledge, all prior studies on the effects of sleep on visual learning employed transient but intrinsically static stimuli. In the present study, binocular disparity changed at a rate of 1/60 s. Reactivations might be unable to track such sudden and arguably non-ecological stimulation conditions. Sleep studies using tasks that rely on static disparity signals will have to be conducted to assess this explanation.

More generally, sleep benefits may depend on the computational principles by which visual performance is improved [38]. For the present task, there are at least three conceivable scenarios: first, training leads to functional changes in early visual processing, which improve the representation of the visual input. This might include a potentiation of the response to the stimulus and associated increases in signal-to-noise ratios. Second, instead of the stimulus representation itself, its read-out by higher-order areas may be improved. This may include strategies such as recruiting neuronal populations with more specific receptive fields or response properties. Third, the information represented in the visual system and its read-out remain equal over training, but the observer optimizes her decision criterion, leading to a better mapping from noisy representations onto one of the two response options (near or far). Diverging neuronal mechanisms underlying the described learning strategies may be critical for subsequent consolidation, such that deeper insights in this area might also inform our understanding on the boundary effects of benefits of sleep.

We conclude that coarse binocular disparity discrimination can be improved via intense training, that these improvements do not generalize to different parts of the visual field, and that neither the improvements nor their generalization are affected by a night of sleep after the task when compared to a day awake. Future studies are needed to investigate whether generalization occurs when tested across the vertical meridian right after the retention period. The absence of sleep effects on a task relying exclusively on differentiating binocular disparities does not exclude that previous inputs are reactivated in the visual system during sleep. Brain areas supporting skill improvements may change over reactivation-mediated consolidation, similar to thalamocortical transfer shown for changes in neuronal responses [14]. Functional imaging studies will have to address this question in the future.

## Supplementary Material

Supplementary Figure 1 Performance development over training

## Data accessibility

Datasets, python scripts and statistical analyses supporting this article have been uploaded to the Open Science Framework (https://osf.io/qj4bh/).
