# Supplementary material for: Binocular disparity-based learning is retinotopically specific and independent of sleep
Source: Philos Trans R Soc Lond B Biol Sci. 2020 Apr 6;375(1799):20190463. doi: 10.1098/rstb.2019.0463 (PMC7209924; doi:10.1098/rstb.2019.0463)
Supplement: Supplementary Figure 1 Performance development over training [file rstb20190463supp1.pdf]

Jens G. Klinzing, Lena Herbrik, Hendrikje Nienborg, Karsten Rauss

Binocular disparity-based learning is retinotopically specific and independent of sleep

Philosophical Transactions of the Royal Society B

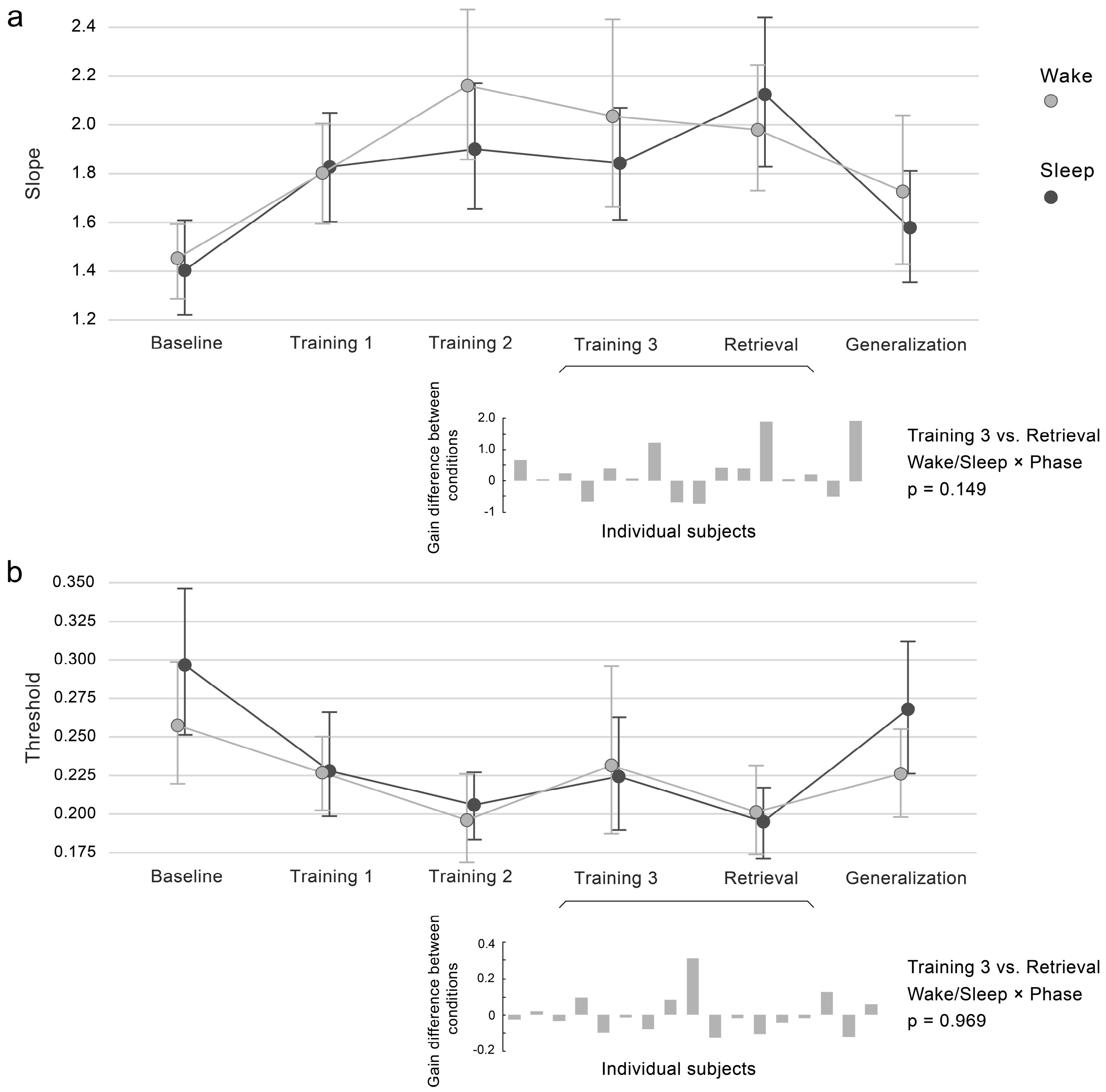

Supplementary Figure 1 Performance development over training. Performance (mean ± 95 % bootstrapped confidence interval) after splitting training trials into three blocks of equal length (Training 1-3). Data are shown for the parameters Slope (a) and Threshold (b) of the psychometric function. In none of the training blocks and for neither parameter did sleep and wake conditions differ significantly (all Holm-corrected p = 1.0). Bars in insets show the difference between conditions in gain (change over retention period; ($Retrieval_{Sleep}$ – Training 3_{Sleep}) – ($Retrieval_{Wake}$ – Training 3_{Wake})) for each subject.

Positive values thus denote higher gain on that parameter in the sleep condition, negative values higher gain in the wake condition. For the parameter Slope, only 16 bars are shown because one of the values from one subject was dropped during outlier rejection. Differential gains over the retention period were analysed directly using a Wake/Sleep × Phase ANOVA including only the last Training block and Retrieval.
